# Negative pressure wound therapy with instillation without open‐window thoracostomy for empyema

**DOI:** 10.1002/rcr2.417

**Published:** 2019-03-19

**Authors:** Kayo Okamoto, Kumiko Matsumoto, Norichika Iga, Seiji Komatsu

**Affiliations:** ^1^ Plastic Surgery Okayama Rosai Hospital Okayama Japan

**Keywords:** Drainage, empyema, instillation, irrigation, negative pressure wound therapy

## Abstract

Treatment of empyema is very challenging. The use of negative pressure wound therapy (NPWT), and NPWT with instillation and dwell time (NPWTi‐d) for wound closure have attracted attention. However, they are both limited to use after open‐window thoracostomy (OWT) performed to control infection. In some patients with poor general conditions, who cannot undergo surgery, no treatment for empyema is available. Therefore, we devised a new treatment for such patients with NPWTi‐d without OWT (non‐OWT NPWTi‐d). Here we present the cases of two patients with refractory empyema after intrathoracic irrigation and drainage, who underwent non‐OWT NPWTi‐d using the fistula of the thoracic drain. Both the patients recovered. The first patient was treated for 31 days. As the empyema persisted, he underwent a repeat intrathoracic drainage after which the wound healed. The second patient was treated for 20 days. Non‐OWT NPWTi‐d may be a new option to treat empyema.

## Introduction

Depending on the stage, various therapeutic options are available for empyema, from thoracic drainage to video‐assisted thoracoscopic surgery (VATS) or open‐window thoracostomy (OWT). After OWT, a muscle flap or free‐flap surgery is often performed for wound closure. These surgical interventions place heavy burdens on patients. Negative pressure wound therapy (NPWT) has attracted attention as it reduces the burden. It promotes wound healing by providing negative pressure to the wound, and many cases of wound closure with NPWT after OWT have been reported 1. NPWT with instillation and dwell time (NPWTi‐d) has been developed for use in infected wounds. NPWTi‐d is used for wound closure after OWT and has shown good results 2. It comprises NPWT coupled with the automated, controlled delivery of a washing solution to the wound bed and the removal of topical wound treatment solutions from it. However, both NPWT and NPWTi‐d have limited use in patients after OWT. Some patients with empyema and poor general conditions cannot undergo OWT. As treatment for such patients is very challenging, developing a new treatment for empyema that can be used without OWT would be very useful. Here, we present a novel treatment for empyema with NPWTi‐d without performing OWT (non‐OWT NPWTi‐d).

## Case Reports

Non‐OWT NPWTi‐d was performed in two patients with empyema at our hospital. A NPWT system, V.A.C. Ulta (KCI USA, Inc.), installed in one of the following two ways, was used. Saline was used as the washing solution. Informed consent was obtained from the patient's relative in the first case and from the patient in the second case.

### Method 1

A thinly processed foam piece was inserted into the fistula, and a port was mounted on the foam.

### Method 2

**Figure 1:**
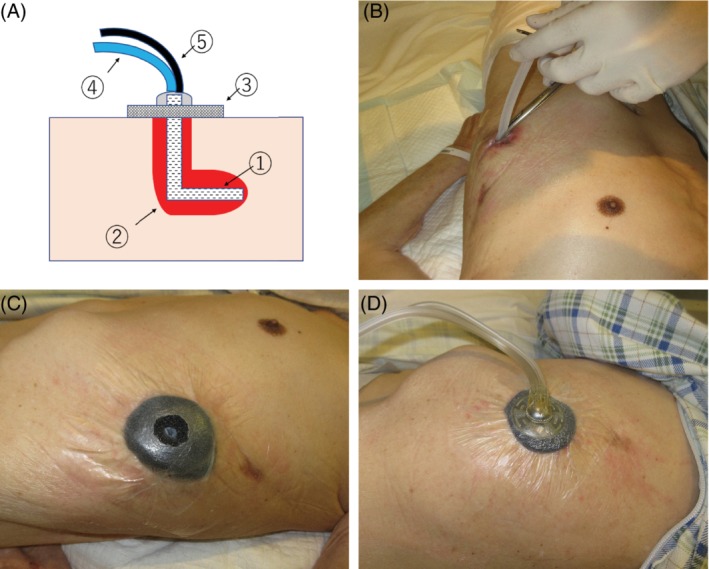
(A) 1: Pleated drain; 2: empyema cavity; 3: foam; 4: fluid injection tube; 5: drainage tube. (B) A pleated drain of 8 mm diameter is inserted into the fistula. (C) The tip of the pleated drain is inserted into the hole of the foam. (D) The fluid injection tube of the port is at the centre of the foam hole so that the liquid injected flows directly into the pleated drain.

The fistula depth was estimated using computed tomography (CT) and forceps. A pleated drain of 8 mm diameter, cut to the same length as the estimated depth, was inserted into the fistula. A foam piece was cut into a circle, with a hole in its centre. The tip of the drain was inserted into the hole. The fluid injection tube of the port was placed at the centre of the hole such that the injected liquid directly entered the drain tube (Fig. 1).

In both methods, the system was changed every 3–4 days. The volume of liquid injected was set to the volume of liquid that flowed into the drainage tube. The system was set such that the liquid was instilled every 3 h, followed by a 10‐min soak time. A negative pressure of −75 mmHg was initially delivered between instillation cycles. It was gradually increased to −125 mmHg. The length of the inserted drain tube was gradually shortened, based on the size of the cavity. When the fistula was considered closed and the drain unnecessary, the port was mounted on the fistula by the foam.

### Case 1

An 80‐year‐old man with post cerebral infarction, depression, epilepsy, gastrostomy, lower limb arteriosclerosis obliterans, and angina pectoris had recurrent empyema in the left thoracic cavity secondary to pulmonary suppuration. He presented with high fever. CT scan revealed empyema, and *Streptococcus constellatus* was detected by wound culture. Irrigation and thoracic drainage (20‐Fr trocar) were performed at the eighth intercostal space in the anterior axillary line, and broad‐spectrum antibiotics were started. VATS decortication could not be performed due to chronic empyema. Thoracic drainage was continued for 21 days; however, the cavity did not noticeably shrink. As the patient could not undergo OWT due to his poor general condition, plastic surgeons were consulted, and on day 24, non‐OWT NPWTi‐d was initiated. Initially, NPWTi‐d was installed as explained in method 1. However, it was changed to method 2 on day 6, in order to more effectively wash the deep part and apply uniform negative pressure. After initiation of non‐OWT NPWTi‐d, the cavity began to shrink. Since NPWTi‐d is indicated for medical insurance only for 28 days in Japan, the patient bears the full expenses if further treatment is continued. Therefore non‐OWT NPWTi‐d was continued for 31 days until the fistula closed. However, three days later, he had a recurrence of high fever. CT scan revealed the recurrence of empyema, and *Staphylococcus aureus* was detected by wound culture. The fistula, which was closed with NPWTi‐d, remained epithelialized. Thoracic drainage (12‐Fr aspiration catheter) was performed under CT guidance, and broad‐spectrum antibiotics were re‐initiated. On day 85, the empyema healed, and he was transferred to another hospital for long‐term care.

### Case 2

**Figure 2:**
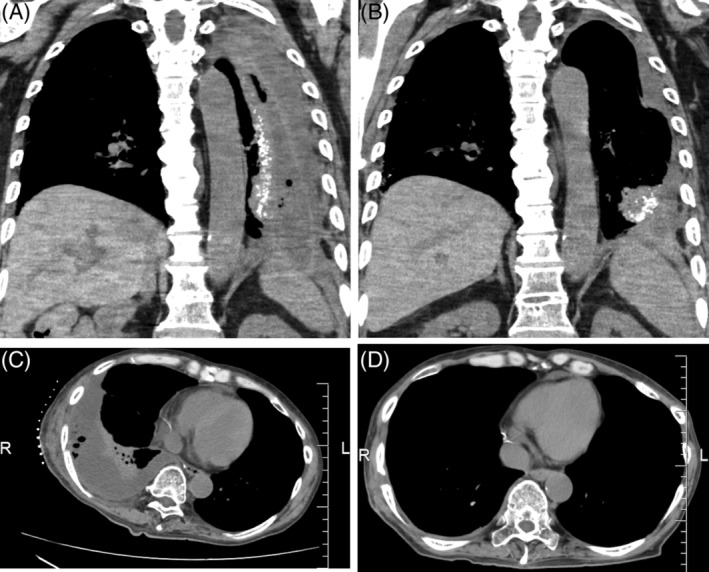
(A) Case 1 (before negative pressure wound therapy with instillation and dwell time without performing open‐window thoracotomy (non‐OWT NPWTi‐d)): An empyema cavity is observed. (B) Case 1 (the day of non‐OWT NPWTi‐d completion): Although the cavity has been confirmed to be reducing in size, the empyema remains. (C) Case 2 (before non‐OWT NPWTi‐d): An empyema cavity is observed. (D) Case 2 (four months after completing non‐OWT NPWTi‐d): No empyema is seen.

A 65‐year‐old man with diabetes, hypertension, and spinal cord injury had empyema in the right thoracic cavity after partial lung resection for adenocarcinoma. He presented with high fever on postoperative day 10. CT scan revealed empyema, and *Streptococcus caprae* was detected by wound culture. Irrigation and thoracic drainage (20‐Fr trocar) were performed under fluoroscopy. Broad‐spectrum antibiotics were started, and treatment was continued for 16 days. Although the cavity was confirmed to be reducing in size, the infection persisted. Hence, plastic surgeons were consulted, and on postoperative day 36, non‐OWT NPWTi‐d was installed by method 2. It was continued for 20 days until the CT scan confirmed that the empyema disappeared. Four months after completing non‐OWT NPWTi‐d, his CT scan showed no empyema recurrence (Fig. 2).

## Discussion

Surgical interventions, including OWT for empyema, place heavy burdens on patients with poor general conditions. Therefore, to reduce the burden on patients, NPWT and NPWTi‐d are being used for treating empyema 1, 2. NPWTi‐d is effective in treating infected wounds. It purifies the thoracic cavity by periodically delivering washing solution into the cavity and applying negative pressure to encourage re‐expansion of the lungs, thereby closing the cavity. To further reduce the burden on patients, mini‐thoracotomy (thoracotomy with a 5–6 cm incision) can be performed. There are some case reports of wound closure using NPWTi‐d after mini‐thoracotomy 3. However, since patients with empyema and poor general conditions cannot undergo surgical interventions and the treatment for such patients is challenging, we sought a novel treatment.

We studied whether NPWTi‐d can be used without OWT and conceived that NPWTi‐d could be applied using the thoracic drain hole. To our knowledge, this is the first report on empyema treatment with NPWT or NPWTi‐d without OWT. Notably, this treatment injects liquid into the thoracic cavity. Therefore, use in patients who exhibit empyema with bronchopleural fistula is contraindicated; however, it is indicated for patients who exhibit empyema with no bronchopleural fistula.

With this treatment, no complications occurred. The following three points were considered: the degree of negative pressure, type of tube, and washing measures used. The negative pressure needs to be sufficient to encourage lung re‐expansion 4. However, there is no unified view about the degree of negative pressure needed to achieve this. In our cases, the pressure was set to −125 mmHg. No obvious respiratory complications occurred, which correlates with past reports suggesting that complications are not expected unless the pressure is extremely high or low.

The type of tube used may be important. We first attempted method 1. However, we were concerned that the negative pressure applied was not uniform and that the deep part of the cavity was not being cleaned by method 1. And we preferred to change to method 2. In method 2, we chose a pleated drain with many side holes to ensure uniform application of negative pressure and washing solution to the cavity. Whether a pleated drain is more effective than a normal drain tube has not been determined by comparative studies. However, the cavity contracted with use of method 2, suggesting that the pleated drain is effective.

We considered the following with regard to washing measures used in our two cases. Although NPWTi‐d is effective for infected wounds, its setting has not yet received a unified view, and more effective methods are being sought. Since there are some reports about the flow of washing solution in NPWTi‐d 5, we considered the possibility that the washing might be made more efficient by changing the position where the tube was inserted. These drain holes were opened without assuming that non‐OWT NPWTi‐d would be used. In case 1, the deep part of the cavity could not be cleaned efficiently due to the position where the drain was inserted. We considered that the washing solution would flow with gravity and decided that inserting the drain in front of and above the cavity would be more effective. However, as the flow of the liquid changes depending on the body position, it may not be effective to have only one insertion site.

We attempted non‐OWT NPWTi‐d for two patients with empyema. For both patients, the empyema healed. Our findings suggest that non‐OWT NPWTi‐d may provide a new treatment option for empyema. Considering that empyema recurred in case 1, a long treatment period may be needed for large empyema or chronic empyema, when treating by non‐OWT NPWTi‐d. However, non‐OWT NPWTi‐d was able to contract a cavity that did not contract with thoracic drainage alone. This treatment is minimally invasive for patients. Therefore, despite the long period of treatment, non‐OWT NPWTi‐d is helpful for patients with refractory empyema who have poor general condition and cannot undergo surgical interventions.

### Disclosure Statement

Appropriate written informed consent was obtained for publication of this case report and accompanying images.
